# Turkish Journal of Hematology: From “Istanbul Contribution to Clinical Science” to “Pubmed Central”

**DOI:** 10.4274/Tjh.31.01

**Published:** 2014-03-05

**Authors:** Hamdi Akan, Bengü Timoçin, İpek Durusu, Aytemiz Gürgey

## ORD. PROF. ERICH FRANK

**Figure 1:**
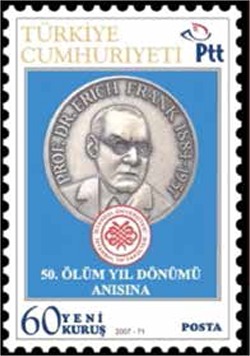
Prof. Dr. Erich Frank (1885-1957)

We respectfully commemorate Prof. Dr. Erich Frank, who pioneered landmark advances in Turkish medicine and established the predecessor of the Turkish Journal of Hematology, on the 130^th^ anniversary of his birth (1884-1957)(Figure 1). Dr. Frank completed his doctorate in Strasburg after graduating from the Breslau University School of Medicine and earned the title of professor in 1919. Oscar Minkowski, who was the most influential among his teachers, directed him toward clinical and experimental medicine. Throughout his life, Dr. Frank evaluated clinical and laboratory studies, defined entities such as essential thrombocytopenia, and led the discovery of synthalin, the precursor of oral anti-diabetics. He was a very well-known and respected scientist on an international scale, receiving an invitation from the United States for the original studies that he conducted. Along with publications related to many other diseases, he concentrated his studies on diabetes, hypertension, and hematologic disorders. He suffered in the early 1930s in Germany as pressure was being applied to people of Jewish descent. Upon recommendations by Mustafa Kemal Atatürk, universities in Turkey were reformed in 1933, ten years after the establishment of the modern Turkish Republic. While Turkish universities were striving to adopt Western standards of learning and a contemporary level of education, scientists of Jewish origin in Europe were simultaneously preparing to leave their countries [1]. Turkey embraced these scientists who were facing persecution at home and work permits were granted to about 100 faculty members and assistants in various scientific branches [2]. Dr. Frank, one such scientist, came to Turkey in 1934 and began to work as director of second Internal Clinic in Istanbul University School of Medicine’s (Vakıf Gureba Hospital). Dr. Frank made much progress in internal medicine during the 23-year period from his arrival in Turkey to his death (1934-1957) [3]. Patients received modern treatment services and clinical research was done. The students that he taught, who later became faculty members themselves, praised Dr. Frank’s courses and legendary conferences. One of Dr. Frank’s most important services was unquestionably the establishment of the medical journal Istanbul Contribution to Clinical Science, first published in 1951 in English, German, and French. The first four volumes were also published in Turkish under the title of Klinik İlmi. The journal initially focused on advancements in internal medicine. After Dr. Frank’s death, during the period in which Prof. Orhan Ulutin was the long-term (1962-2002) editor, studies began to be published with a primary focus on the hematologic sciences. Prof. Ulutin (1924-2011) worked in Frank’s clinic and laboratory. He was interested in hematology, particularly qualitative platelet disorders and coagulation factors, and he founded the Department of Hematology in the Internal Clinic of Istanbul University in 1963. Ulutin and his colleagues established the Turkish Society of Hematology in 1967. The journal then became the official publication of that association in 1971, under the name of New Istanbul Contribution to Clinical Science. It was indexed in Index Medicus between 1965 and 1982 until the last volume, Volume 13 [4]. The name of the journal was changed to Turkish Journal of Hematology in 1995. 

Dr. Frank was committed to Turkey during his lifetime and he became a Turkish citizen. After the Second World War, he received brilliant offers from American and German universities because of his scientific capability and creativity, but he declined these opportunities, stating that: “While I was experiencing the bitter astonishment of being thrown out from my country, only Turkey opened its arms and embraced me. This is my country, and I cannot show ingratitude”. Dr. Frank was buried with a state funeral in the Istanbul Aşiyan Cemetery according to his wishes after he passed away in 1957. Prof. Dr. Orhan Ulutin, who had been Dr. Frank’s assistant, gave a conference in 2006 entitled “The Place of Prof. Dr. Erich Frank in the World of Science and His Contributions to Turkish Medicine” and then prepared a book including other information about Dr. Frank [5]. 

**THE JOURNAL NOW**

The Turkish Journal of Hematology (TJH) is published quarterly by the Turkish Society of Hematology. It is an independent, non-profit, peer-reviewed international English-language periodical encompassing subjects relevant to hematology. 

The Editorial Board of TJH adheres to the principles of the World Association of Medical Editors (WAME), International Council of Medical Journal Editors (ICMJE), Committee on Publication Ethics (COPE), Consolidated Standards of Reporting Trials (CONSORT), and Strengthening the Reporting of Observational Studies in Epidemiology (STROBE). 

The aim of TJH is to publish original hematological research of the highest scientific quality and clinical relevance. Additionally, educational material, reviews on basic developments, editorial short notes, case reports, images in hematology, and letters from hematology specialists and clinicians covering their experience and comments on hematology and related medical fields as well as social subjects are published. 

**TJH is indexed as follows:** (Table 1 and Table 2)

• PubMed Central (August 2013)

• ProQuest (2010)

• Science Citation Index Expanded (March 2009)

• CINAHL (2008)

• Gale/Cengage Learning (2008)

• EBSCO (2008)

• DOAJ (2008)

• TÜBİTAK/ULAKBİM Turkish Medical Database (2008)

• Scopus (2007)

• EMBASE (1999)

• Index Copernicus (1999)

The journal is published by Galenos and online manuscript submission is done via the Thomson-Reuters Scholar One system.

The journal has a wide readership and currently receives manuscripts from roughly 40 different countries around the world. The total number of reviewers in 2013 was 3355. Past and current editors are:

1951-1957: Prof. Dr. Erich Frank

1962-2002: Prof. Dr. Orhan Ulutin

2002-2005: Prof. Dr. Hamdi Akan

2006-present: Prof. Dr. Aytemiz Gurgey 

**Turkish Journal of Hematology and Open Access**

Since the Internet has become a cornerstone of academic life, new concepts have accordingly been introduced to our daily life, such as “open access”. Enormous amounts of information that we never had the chance to reach in the past can now be accessed immediately, and this has brought about new opportunities in medical journalism. Open access is not only a concept but also a movement. Open access in terms of literature is defined as “free availability on the public internet, permitting any users to read, download, copy, distribute, print, search, or link to the full texts of these articles, crawl them for indexing, pass them as data to software, or use them for any other lawful purpose, without financial, legal, or technical barriers other than those inseparable from gaining access to the internet itself” [6]. This movement first started in Budapest, followed by Bethesda and Berlin. Although the open access movement also covers music and book publishing, these areas depend on their financial structures, limiting their borders of open access. While these industries can only survive by financial expansion, this is not the case for medical journals. Most of the time, the only expense related to online publishing is limited to the server cost, domain name, and salaries for personnel. The automation in on-line publishing makes the process very easy and very cost-effective, reducing manpower and time. The number of journals available with open access is increasing rapidly and the availability of gold and green open access copies by scientific discipline is shown in Table 3 [7].

Open access brought about radical changes in medical journals. The availability of medical journals on the Internet made it possible to reach contents directly from the web pages of the journals, from the domains of publishing companies such as Springer Link or Elsevier, or from journal depositories. In this way, the limited number of readers of a journal increased exponentially. The most important improvement in this area was the addition of PubMed Central (PMC) to the National Library of Medicine; this index became the main repository for journals published online [8]. PMC was launched in 2000 and is a free archive of biomedical and life sciences journal literature. Although the standard criteria for indexing medical journals are also valid here, an additional requirement is the sending of the articles in a required format (mostly XML or SGML). 

The Directory of Open Access Journals (DOAJ) is a good example of such repositories [9]. Not only PubMed but also other indexes are covered here. If we look at the number of journals in the DOAJ according to the country of origin, we see that Turkey is the 12^th^ country on the list (Table 4) [9]. Interestingly, Iran is the 11th country on the list. This can be explained by the fact that all Iranian biomedical journals (n=163) have an open access mode [10]. In South Korea, one-third of the open access medical journals are archived in PMC [9]. Some privileged journals such as the BMJ and PLOS have a very significant online impact. Although a journal being indexed in PMC does not necessarily mean that it is also indexed in Medline, all PMC journals can be reached in PubMed. The reason for this is that PubMed covers: 1) MEDLINE indexed journals, 2) journals/manuscripts deposited in PMC, and 3) the NCBI Bookshelf [6].

During the transition of TJH to an international journal in the 1990s, main targets were to become an open access journal and to be indexed in the major databases. The first target was achieved in a short time, followed by indexing in various indexes such as the Science Citation Index Expanded, EMBASE, Scopus, CINAHL, Gale/Cengage Learning, EBSCO, DOAJ, ProQuest, Index Copernicus, and the TÜBİTAK/ULAKBİM Turkish Medical Database [11]. During this process, TJH was one of the first medical journals in Turkey to publish on the Internet with the full text of articles available to readers. 

TJH made its first application to PMC immediately in 2000, as soon as PMC was launched. At that time, technical difficulties obstructed the path, but in 2013, TJH became indexed in PMC. TJH is the third journal indexed from Turkey and this number is increasing. Although this is a good achievement, compared to countries such as South Korea, India, or Iran, there is still a long way to go. One-third of the open access medical journals in South Korea are also indexed in PMC. This is also true for India, where nearly all biomedical journals are open access and some have found their way into PMC [10]. Supporting open access journalism is also an accepted strategy in developed countries such as the United Kingdom. In the UK, the government adopted a national strategy in 2012 that supports research funds for open access publishing.

Despite these achievements, there are also problems with open access journals. The main criticism is directed toward the peer-review process. A recent paper published in the 4 October 2013 volume of Science [12] underlines one such important problem. Three hundred and four versions of a fake, non-existent study were sent to open access journals and nearly half of these peer-review journals accepted the study with minor or no revisions. This shows that as the stress of publishing online journals increases, the quality of the peer-review process decreases. Of course, this problem may also exist for non-open access journals. Another problem in open access journalism is the fee charged for manuscripts to be published. It looks as if the pressure on medical doctors for publishing new articles opened an avenue for financial benefit among companies that own or host open access journals.

In the medical sciences, open access is a revolutionary approach to sharing and distributing research, development, and innovation, provided that strict rules for quality control are maintained all throughout the process. TJH now fulfills this requirement and is proud to be a member of the open access community.

## Figures and Tables

**Table 1:**
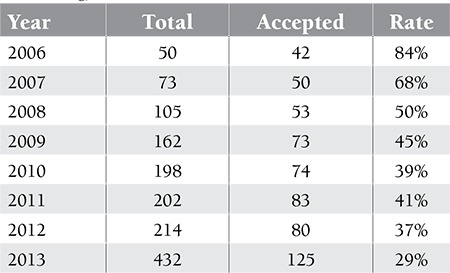
Manuscripts submitted to the Turkish Journal of Hematology

**Table 2:**
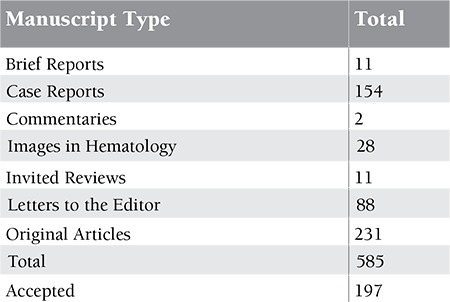
Details of the manuscripts submitted and published between January 2012 and November 2013 (2 years)

**Table 3:**
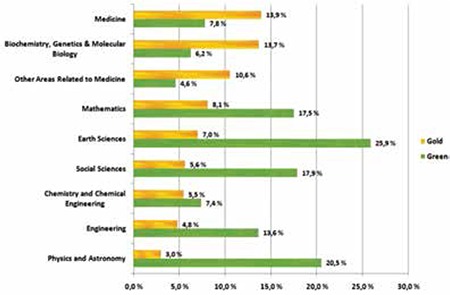
Publication bias of included nested case-control studies analysed by funnel plot

**Table 4:**
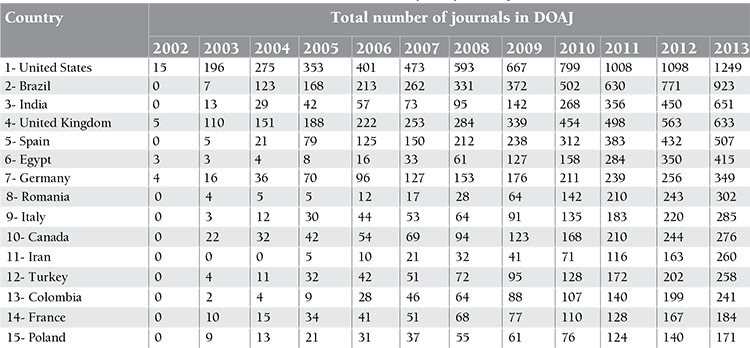
Publication bias of included nested case-control studies analysed by funnel plot
